# P-1063. SCY-247: A Second-generation IV/Oral Triterpenoid Antifungal with Extensive Tissue Distribution and Pharmacokinetics, and Low Drug-Drug Interaction Potential

**DOI:** 10.1093/ofid/ofae631.1252

**Published:** 2025-01-29

**Authors:** Steve Wring, Katyna Borroto-Esoda, Nathan P Wiederhold, David A Angulo

**Affiliations:** SCYNEXIS, Inc., Jersey City, New Jersey; SCYNEXIS, Inc., Jersey City, New Jersey; University of Texas Health San Antonio, San Antonio, TX; SCYNEXIS, Inc., Jersey City, New Jersey

## Abstract

**Background:**

SCY-247 is an IV/oral second-generation triterpenoid anti-fungal for systemic fungal infections including multidrug resistant pathogens. Here we present translational data for nonclinical pharmacokinetics (PK) including dose-related tissue disposition in a murine *C. glabrata* model and correlation to reductions in fungal burden. Further, *in vitro* CYP450 interactions were evaluated to assess potential for clinical drug-drug interactions (DDIs).

**Methods:**

The PK of SCY-247 was determined in rats and dogs. Plasma and tissue exposures were compared to reductions in kidney and lung fungal burden in a model of invasive *C. glabrata*. Neutropenic mice were infected IV with *C. glabrata* (∼10^8^ yeast cells/mouse) and sacrificed on Day +7. Potential for inhibiting or inducing CYP450 isoforms was assessed *in vitro* using methods supportive of an FDA Investigational New Drug (IND) Application.

**Results:**

SCY-247 has low clearance (∼10% liver blood flow) with half-life ≥15 hours following oral and IV administration to rat and dog consistent with once-daily administration in humans. Volume of distribution is ∼3 L/kg supportive of extensive tissue distribution. Exposure increased on repeat administration. Solubility of oral SCY-247 as a salt is >30 mg/mL at gastric pH consistent with rapid dissolution in the stomach and rapid absorption - as demonstrated by an early Tmax (0.5-2hr) following oral administration. In a murine model of *C. glabrata* infection, SCY-247 demonstrated dose-related increases in exposure and corresponding reduction in fungal burden (right figure panel) with higher distribution in kidney and lung tissue compared to plasma (left figure panel). *In vitro*, SCY-247 did not induce human CYP450s 1A2, 2B6 or 3A4. The IC_50_ values for inhibition of human 2C9, 2C19 and 3A isoforms were >20 μM ( >10-fold higher than the anticipated clinical Cmax) supportive of low risk of DDI’s for these isoforms.

**Conclusion:**

SCY-247 has demonstrated dose- and tissue-related exposure reductions in fungal burden, PK consistent with once daily dosing to humans and low risk for CYP450-related DDIs.

**Disclosures:**

**Steve Wring, PhD**, SCYNEXIS, Inc.: Advisor/Consultant **Katyna Borroto-Esoda, MS**, Scynexis Inc: Advisor/Consultant **Nathan P. Wiederhold, PharmD**, BioMerieux: Grant/Research Support|F2G: Advisor/Consultant|F2G: Grant/Research Support|Mycovia: Grant/Research Support|Scynexis: Grant/Research Support|Sfunga: Grant/Research Support **David A. Angulo, MD**, SCYNEXIS, Inc.: Board Member|SCYNEXIS, Inc.: SCYNEXIS, INC Officer and Board Member, SCYNEXIS Patents|SCYNEXIS, Inc.: Stocks/Bonds (Public Company)

